# Intratumoral Virus-Like Particles Containing a TLR9 Agonist Combined with Systemic αPD-1 Activate Tumor-Specific CD8^+^ T Cells

**DOI:** 10.1158/2767-9764.CRC-26-0175

**Published:** 2026-05-01

**Authors:** Travis D. Fischer, Caitlin D. Lemke-Miltner, George J. Weiner

**Affiliations:** 1 Cancer Biology Graduate Program, University of Iowa, Iowa City, Iowa.; 2 Holden Comprehensive Cancer Center, University of Iowa, Iowa City, Iowa.; 3 Department of Internal Medicine, University of Iowa, Iowa City, Iowa.

## Abstract

One strategy for enhancing the anticancer immune response is to inject tumors with immunostimulatory agents that modify the tumor microenvironment (TME) to induce a stronger antitumor T-cell response. *In situ* immunization with vidutolimod (Vidu), a virus-like particle containing a CpG-A TLR9 agonist, has demonstrated antitumor activity in preclinical and early-phase clinical studies; however its effect on tumor-specific CD8^+^ T cells remain poorly defined. Using the OT-1 model, we assessed how Vidu affects the activation, cytotoxicity, and antitumor activity of tumor-specific CD8^+^ T cells *in vitro* and *in vivo*. *In vitro*, Vidu reduced proliferation but increased expression of both activation and exhaustion markers. *In vivo*, repeated intratumoral Vidu injections induced a transient increase in the frequency of intratumoral tumor-specific CD8^+^ T cells and enhanced antitumor activity. The addition of αPD-1 to Vidu led to a persistent increase in intratumoral tumor-specific CD8^+^ T cells and sustained tumor control. Vidu treatment increased expression of markers associated with terminal exhaustion on intratumoral tumor-specific CD8^+^ T cells. Such treatment also increased the number of circulating tumor-specific CD8^+^ T cells that expressed high PD-1 but lacked coexpression of other exhaustion markers. Together, these findings demonstrate that Vidu treatment expands the number of intratumoral and circulating tumor-specific CD8^+^ T cells and that the number of tumor-specific CD8^+^ T cells and the antitumor response is sustained by the addition of αPD-1. These results support continued evaluation of Vidu as a cancer immunotherapeutic agent, including in combination with immune checkpoint blockade.

**Significance:**

*In situ* immunization with Vidu combined with αPD-1 therapy enhances the antitumor response by tumor-specific CD8^+^ T cells by expanding the systemic and intratumoral populations. These findings demonstrate how Vidu alters T-cell biology and supports its continued development, particularly in combination with checkpoint blockade to optimize antitumor immunity.

## Introduction

Cancer treatment with αPD-1/PD-L1 immune checkpoint blockade (ICB) has been a major clinical advance for patients with a variety of cancers (1–3). However, a majority of patients do not respond to such immunotherapy, and some that initially respond to treatment develop resistance (4). Resistance in some cases is associated with tumors that lack T-cell infiltration and interferon (IFN) signatures (5, 6). One approach to overcoming such resistance is to modify the tumor microenvironment (TME) through direct intratumoral (IT) injection of immunostimulatory agents in a process known as *in situ* immunization (7). Among agents used for *in situ* immunization are Toll-like receptor (TLR) agonists. CpG oligodeoxynucleotides (ODN) are TLR9 agonists that mimic unmethylated bacterial DNA (8). In humans, TLR9 expression is almost exclusively restricted to B cells and plasmacytoid dendritic cells (pDC), making them targets for TLR9 agonist–based therapies (8, 9). Class-A CpG ODN TLR9 agonists are potent inducers of IFNα production by pDCs but are highly susceptible to nuclease degradation, which has limited *in vivo* evaluation (8).

To overcome the rapid degradation of CpG-A, vidutolimod (Vidu) was developed. Vidu is a nonreplicative virus-like particle (VLP) composed of CpG-A surrounded by a Qβ bacteriophage capsid that protects the CpG-A from nuclease degradation. Uptake of Vidu by pDCs, key cells in the response to Vidu, is dependent on the coating of Vidu with anti-Qβ antibodies (αQβ) which are generated *in vivo* by an initial exposure to Vidu (9, 10).We recently reported that the mechanism of uptake of Vidu/αQβ immune complexes is mediated by their binding to BDCA2, a pDC-exclusive receptor (11). Uptake of Vidu by pDCs via BDCA2 leads to the delivery of the CpG payload to TLR9 and subsequent production of IFNα (8). Vidu has demonstrated modest but clear clinical activity as a single agent, and preliminary evidence suggests that clinical responses to Vidu are increased when combined with αPD-1 (12–14). In a clinical trial in PD-1–refractory melanoma, Vidu combined with αPD-1 therapy resulted in a clinical response in 25% of patients, with the median duration of response being 25.6 months (15). In preclinical studies, the therapeutic response to Vidu therapy was lost when CD4^+^, CD8^+^, or both T-cell subsets were depleted (9).

CD8^+^ T-cell antitumor immunity can be suppressed by chronic exposure to antigen that results in a state of functional impairment and dysfunction known as T-cell exhaustion (16–18). There is ongoing discussion concerning how best to define T-cell exhaustion and the relationship between the phenotype and function of such cells. There is general consensus that exhausted T cells tend to coexpress higher levels of inhibitory receptors (e.g., PD-1, CTLA-4, LAG3, TIGIT, and TIM3), produce fewer inflammatory cytokines (e.g., IFNγ, TNFα, and IL2), and have decreased proliferative capacity (19–22). Progenitor-exhausted T cells represent a category of exhausted T cells still capable of responding to infections and tumors. These cells produce high levels of inflammatory cytokines and have robust proliferation upon restimulation but also express inhibitory receptors, including CTLA-4, LAG3, and TIGIT but not TIM3 (22). Terminally exhausted T cells represent a deeper state of T-cell exhaustion. Terminally exhausted T cells produce lower levels of inflammatory cytokines, have minimal proliferation upon restimulation, and are marked by higher, chronic expression of multiple inhibitory receptors, especially TIM3 (22–24). Progenitor-exhausted T cells are able of reinvigoration by ICB therapy, but terminally exhausted T cells are not (22–24).

Understanding how *in situ* immunization with Vidu affects the number, activation, and exhaustion of tumor-specific T cells in the tumor and systemically will contribute to our ability to advance *in situ* immunization, including combination treatments, and to develop superior agents capable of enhancing and maintaining the antitumor T-cell response. Studies to date exploring the impact on T cells of *in situ* immunization with Vidu, with and without αPD-1 therapy, have involved evaluation of the overall T-cell population. In this study, we examine how treatment with Vidu affects tumor-specific CD8^+^ T-cell activation and exhaustion *in vitro* and *in vivo* in both the tumor and the circulation.

## Materials and Methods

### Mice

Female wild-type (WT) C57BL/6 (strain #: 000664, RRID: IMSR_JAX:000664) and OT-1 C57BL/6 (strain #: 003831, RRID: IMSR_JAX:003831) mice ages 6 to 8 weeks were purchased from The Jackson Laboratory and maintained in animal facilities at the University of Iowa, Iowa City, Iowa. Female C57BL/6 mice were used exclusively to minimize variability from sex-specific immune and hormonal differences, which are well-documented to influence tumor growth and immune responses in this strain. Animal housing and experimental procedures were approved by the University of Iowa Institutional Animal Care and Use Committee (animal protocol #3121236).

### Cell lines and culture medium

EL4 (cat. #TIB-39, RRID: CVCL_0255) and E.G7-OVA (cat. #CRL-2113, RRID: CVCL_3505) cell lines were obtained from the American Type Culture Collection and stored in liquid nitrogen tanks in the Weiner Laboratory. All cells were cultured in RPMI 1640 (Thermo Fisher Scientific, cat. #11875093) supplemented with 10% fetal bovine serum, 1× GlutaMAX (Thermo Fisher Scientific, cat. #35050-061), 1 mmol/L sodium pyruvate (Thermo Fisher Scientific, cat. #11360-070), 10 mmol/L HEPES (Thermo Fisher Scientific, cat. #15630-080), 50 μmol/L 2-mercatoethanol (Sigma-Aldrich, cat. #M-6250), and 50 μg/mL gentamycin (IBI Scientific, cat. #IB02030) at 37°C in a humidified atmosphere with 5% CO_2_. E.G7-OVA tumor cells were cultured with supplemental G418 disulfate solution (IBI Scientific, cat. #IB02060), 1 mg/mL, to ensure OVA:MHC class I expression. Cell lines used for culture were passaged no more than 20 times and were grown for no more than 2 weeks after thawing. EL4 and E.G7-OVA cell lines were authenticated and confirmed to be *Mycoplasma*-negative (IDEXX BioAnalytics).

### Vidu and αQβ

Vidu was provided by Checkmate Pharmaceuticals (now Regeneron). A murine IgG1 monoclonal antibody specific for the Qβ capsid of Vidu was generated in collaboration with the Iowa State University Protein Facility. Vidu was used at a final concentration of 5 μg/mL based on protein, and αQβ antibodies were used at a final concentration of 5 μg/mL.

### *In vitro* culture conditions

Spleens were collected from C57BL/6 OT-1 mice and dissociated in MACs C tubes (Miltenyi) into single cells. Single cells were strained through a 70-μm strainer (Fisherbrand), and red blood cells were removed via RBC Lysis Buffer (BioLegend, cat. #420302). Unfractionated OT-1 splenocytes (2 × 10^5^ per well) were plated in 96-well U-bottom plates and were cultured with nothing, E.G7-OVA tumor cells (50:1 splenocyte to tumor cell ratio), or SIINFEKL peptide (10 ng/mL, Anaspec, cat. #AS-60193). After 1 hour of stimulation, cells were treated with Vidu (5 μg/mL) and αQβ antibodies (5 μg/mL). Short-term cultures to determine T-cell activation and proliferation were analyzed after 3 days.

Long-term cultures were utilized to determine the development of T-cell exhaustion after 7 and 14 days. Cells were stimulated on day 0 (Single SIINFEKL dose) or daily on days 0 to 4 (Repeat SIINFEKL dose) with SIINFEKL peptide (10 ng/mL) followed by the addition of Vidu/αQβ (5 μg/mL each). Cells were analyzed via flow cytometry on days 7 and 14 for activation and inhibitory marker expression. Surface marker expression was determined on days 7 and 14. Inflammatory cytokine expression was evaluated following restimulation with additional SIINFEKL (10 ng/mL) 24 hours (days 6 and 13, respectively) before evaluation on days 7 and 14. Media were changed every 3 days with supplemental IL7 (5 ng/mL, Peprotech, cat. #217-17) and IL15 (5 ng/mL, Peprotech, cat. #210-15).

### Flow cytometry

Splenocytes were evaluated by flow cytometry for surface and intracellular markers. Following culture, cells were treated with brefeldin A (1×, BioLegend, cat. #420601) 4 to 6 hours prior to evaluation. Cells were collected and stained with Zombie Aqua Fixable Viability dye (BioLegend, cat. #423101) or Live/Dead Blue Viability dye (Thermo Fisher Scientific, cat. #L23105) according to the manufacturers’ protocols to distinguish live from dead cells. To identify and characterize OT-1 CD8^+^ T cells, samples were stained with SIINFEKL tetramer H-2Kb SIINFEKL class I MHC (Kb -OVA_257_; PE, NIH Tetramer Core Facility), followed by antibodies against CD45 (clone 30-F11, Spark Blue 550, BioLegend, cat. #103165, RRID: AB_2819791), CD3 (clone 17A2, BV750, BioLegend, cat. #100249, RRID: AB_2734148), CD335 (clone 29A1.4, BV605, BioLegend, cat. #137619, RRID: AB_2562452), CD172a (cone P84, APC, BioLegend, cat. #144013, RRID: AB_2564060), LAG3 (clone C9B7W, BV650, BioLegend, cat. #125227, RRID: AB_2687209), PD-1 (clone 29F.1A12, BV785, BioLegend, cat. #135225, RRID: AB_2563680), CD25 (clone PC61, PerCP, BioLegend, cat. #102028, RRID: AB_2295974), TIGIT (clone 1G9, PE/Dazzle 594, BioLegend, cat. #142110, RRID: AB_2566573), TIM3 (clone RMT3-23, PE/Cyanine7, BioLegend, cat. #119716, RRID: AB_2571933), CTLA-4 (UC10-4B9, PE/Fire 810, BioLegend, cat. #106335, RRID: AB_2941377), CXCR3 (clone CXCR3-173, APC/Fire 750, BioLegend, cat. #126539, RRID: AB_2650829), CD8 (clone KT15, FITC, Abcam, cat. #ab22504, RRID: AB_447109), CD4 (clone GK1.5, BUV496, BD Biosciences, cat. #612952, RRID: AB_2813886), and CD19 (clone 1D3, BUV661, BD Biosciences, cat. #612971, RRID: AB_2870243). To detect intracellular/nuclear proteins, samples were fixed and permeabilized using eBioscience Foxp3 Fixation/Permeabilization Kit (Thermo Fisher Scientific) and stained with antibodies against IFNγ (clone XMG1.2, BV421, BioLegend, cat. #505829, RRID: AB_10897937), TNFα (clone MP6-XT22, Alexa Fluor 700, BioLegend, cat. #506338, RRID: AB_2562918), and granzyme B (clone QA16A02, PerCP/Cyanine5.5, BioLegend, cat. #372211, RRID: AB_2728378). For proliferation assays, whole OT-1 splenocytes were labeled with CellTrace Violet (Invitrogen, cat. #C34557) before plating. Cells were evaluated using a Cytek Aurora Spectral Flow Cytometer (University of Iowa Flow Cytometry Facility, RRID: SCR_019826), and data were analyzed using FlowJo v10 (BD Biosciences, RRID: SCR_008520). Flow cytometry gating strategy is provided in Supplementary Fig. S1.

### Granzyme B ELISA

OT-1 splenocytes (2 × 10^5^ per well) were cultured in 96-well U-bottom plates with treatments for 3 days. Granzyme B in the supernatant was measured by ELISA (Thermo Fisher Scientific, cat. #88-8022-88) according to the manufacturer’s protocol. ELISA plates were read on a ThermoMax Precision microplate reader using SoftMax microplate data acquisition software (Molecular Devices, RRID: SCR_014240).

### Cytotoxicity assay

Unfractionated OT-1 splenocytes were stimulated with SIINFEKL peptide (10 ng/mL) with Vidu and αQβ (5 μg/mL each) added 1 hour later. After 3 days of culture, untouched CD8^+^ T cells were isolated for use as effector cells using a CD8α^+^ T-cell isolation kit (Miltenyi, cat. #130-104-075). E.G7-OVA tumor cells were used as target cells. E.G7-OVA cells were treated with mitomycin C (1 μg/mL) for 2 hours and washed 3× with R10 media. E.G7-OVA cells were then labeled with CellTrace Violet (1 μL/mL for every 10^6^ cells).

OT-1 CD8^+^ T cells (effectors) were combined with labeled E.G7-OVA cells (targets) at various ratios and incubated for 18 hours. After 18 hours, cells were stained and analyzed by multispectral flow cytometry. Viable E.G7-OVA cells were characterized as Live/Dead blue^-^ CD45^+^ CD3^+^ CellTrace Violet^+^. The absolute count of viable E.G7-OVA cells and %cytotoxicity was determined by the following formula: %cytotoxicity = [(number of viable target cells/mL in control − number of viable target cells/mL in treated samples) ÷ (number of viable target cells/mL in control)] × 100.

### *In vivo* analysis

#### Vidu as a monotherapy

Day 0 was defined as the first day of Vidu treatment. WT C57BL/6 mice were injected subcutaneously on day −27 with 50 μg Vidu to induce generation of αQβ antibodies as previously reported (9). On day −13, 10^6^ E.G7-OVA tumor cells were implanted into the left flank. OT-1 splenocytes corresponding to 10^4^ OT-1 CD8^+^ T cells were adoptively transferred into tumor-bearing mice by retro-orbital injection on day −3. IT injections of saline or Vidu (400 μg based on protein, 100 μg based on ODN) were administered on days 0, 4, and 8. Vidu was diluted to a final volume of 100 μL per mouse using saline. Serial fine-needle aspirates (FNA) and blood sampling were performed as indicated, and cells were stained for flow analysis. Tumor measurements and survival were monitored out to 100 days.

#### Combination Vidu and αPD-1

Day 0 was defined as the first day of Vidu treatment. WT C57BL/6 mice were injected subcutaneously on day −31 with 50 μg Vidu to induce generation of αQβ antibodies. On day −12, 10^6^ E.G7-OVA tumor cells were implanted into the left flank. Serial tumor measurements were collected. OT-1 splenocytes corresponding to 10^4^ OT-1 CD8^+^ T cells were adoptively transferred into tumor-bearing mice by retro-orbital injection on day −1. IT injections of saline or Vidu (400 μg based on protein, 100 μg based on ODN) were administered on days 0, 3, and 6. Vidu was diluted to a final volume of 100 μL per mouse using saline. Isotype IgG2aκ (175 μg, Bio X Cell, clone: 2A3, cat. #BE0089, RRID: AB_1107769) and αPD-1 IgG2aκ (175 μg, Bio X Cell, clone: RMP1-14, cat. #BE0146, RRID: AB_10949053) were diluted to a final volume of 100 μL per mouse using InVivoPure pH 7.0 Dilution Buffer (Bio X Cell, cat. #IP0070) and injected intraperitoneally immediately following IT injections. An additional intraperitoneal treatment was given on day 9. Mice were sacrificed on day 7 (24 hours after the final Vidu treatment) or day 10 (4 days after the final Vidu treatment). Blood, tumor, draining lymph node (DLN), and spleen were collected for flow analysis. Tumors from mice were weighed and used for flow analysis.

### Statistical analysis

Statistical analyses were performed in GraphPad Prism (v10.2.2, RRID: SCR_002798). All data are presented as mean + SEM. Paired or unpaired student *t* tests were used to compare two groups. One-way ANOVA with Tukey test for multiple comparisons was used to compare the means of three or more independent (unrelated) groups. Two-way ANOVA with Sidak multiple comparisons test was used for comparisons of more than two groups, as noted in figure legends. A Kaplan–Meyer curve with log-ranked (Mantel–Willcox) test was generated to determine survival significance. Data were considered significant when *P* values ≤ 0.05 (*, *P* ≤ 0.05; **, *P* ≤ 0.01; ***, *P* ≤ 0.001; ****, *P* ≤ 0.0001). Data were considered not significant (ns) when *P* values > 0.05.

## Results

### Treatment with Vidu increases activation marker expression by tumor-specific CD8^+^ T cells

We previously demonstrated that the therapeutic response to *in situ* immunization with Vidu in mouse models is dependent on both CD4^+^ and CD8^+^ T cells (9). However, Vidu did not significantly increase the number of overall IT CD4^+^ or CD8^+^ T cells. To further investigate the impact of Vidu on tumor-specific T cells, we utilized the well-established transgenic OT-1 model in C57BL/6 mice in which all CD8^+^ T cells express a T-cell receptor specific for the immunodominant ovalbumin peptide SIINFEKL. Given that response to Vidu is dependent on the presence of antibodies against the Qβ capsid of Vidu, αQβ was added along with Vidu in all *in vitro* samples when evaluating the impact of Vidu.

**Figure 1.**
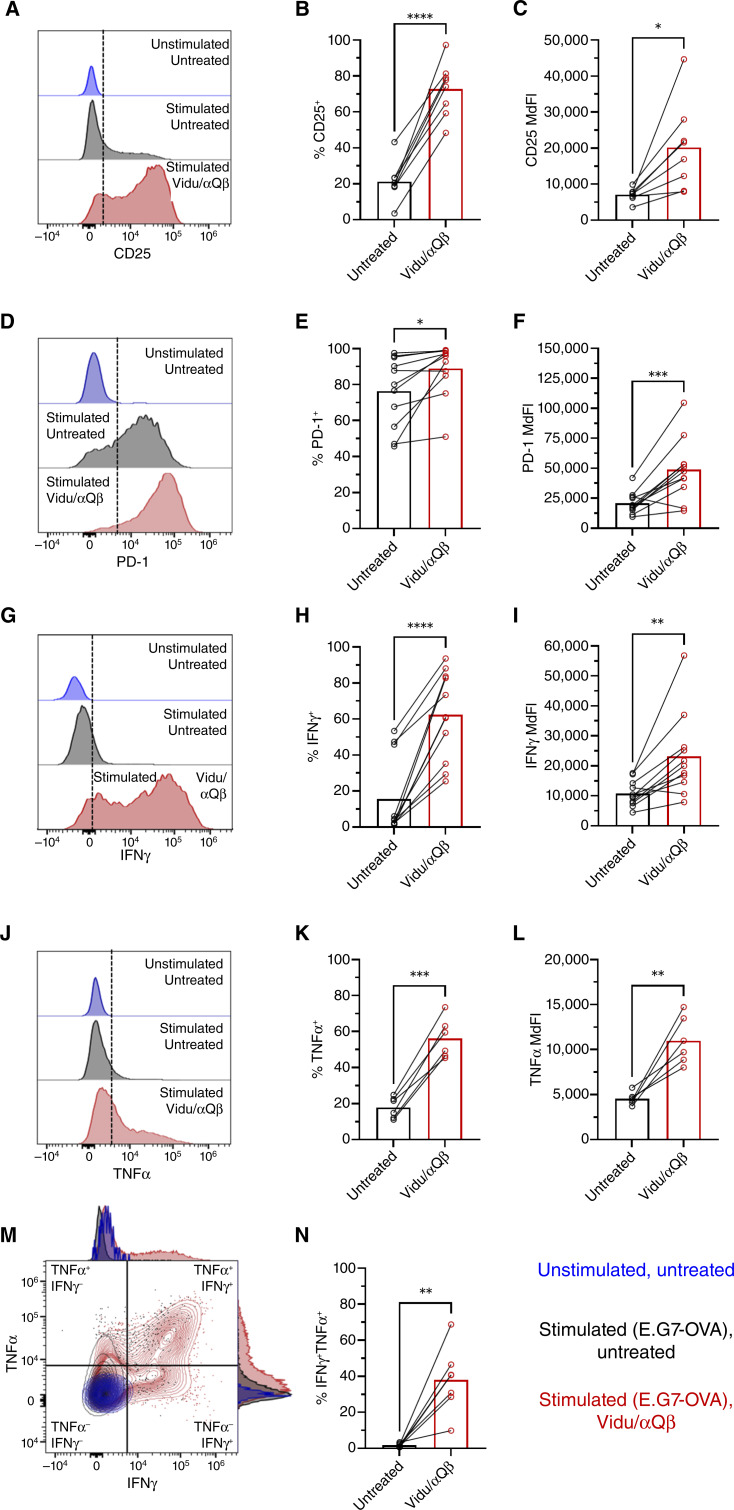
Vidu increases activation marker expression by tumor-specific CD8^+^ T cells. OT-1 splenocytes were cultured with either media (unstimulated) or E.G7-OVA cells (stimulated) for 1 hour, followed by no additional treatment (untreated) or the addition of Vidu and αQβ (treated). After 3 days of coculture, activation marker expression by OT-1 CD8^+^ T cells was analyzed by multicolor spectral flow cytometry. **A,** Representative histograms of CD25 expression on OT-1 CD8^+^ T cells. **B,** Frequency of CD25^+^ OT-1 CD8^+^ T cells and (**C**) MdFI of CD25 expression (*n* = 8 mice/group) in untreated (black) and treated (red) samples. **D,** Representative histograms of PD-1 expression on OT-1 CD8^+^ T cells. **E,** Frequency of PD-1^+^ OT-1 CD8^+^ T cells and (**F**) MdFI of PD-1 expression (*n* = 11 mice/group). **G,** Representative histograms of intracellular IFNγ expression by OT-1 CD8^+^ T cells. **H,** Frequency of IFNγ^+^ OT-1 CD8^+^ T cells and (**I**) MdFI of IFNγ expression (*n* = 11 mice/group). **J,** Representative histograms of intracellular TNFα expression by OT-1 CD8^+^ T cells. **K,** Frequency of TNFα ^+^ OT-1 CD8^+^ T cells and (**L**) MdFI of TNFα expression (*n* = 6 mice/group). **M,** Representative histogram and dot plot and (**N**) frequency of polyfunctional IFNγ^+^TNFα^+^ OT-1 CD8^+^ T cells (*n* = 7 mice/group). Each symbol connected by a line represents an individual mouse in untreated and Vidu/αQβ conditions and the mean of the group, with error bars indicating the SEM. Statistical significance was determined using a paired *t* test: *, *P* < 0.05; **, *P* < 0.01; ***, *P* < 0.001; ****, *P* < 0.0001.

Unfractionated OT-1 splenocytes, which contain antigen-presenting cells, pDCs, and OT-1 CD8^+^ T cells, were cocultured with E.G7-OVA tumor cells, followed by the addition of Vidu/αQβ after 1 hour. The activation status of OT-1 CD8^+^ T cells, as indicated by surface CD25 and PD-1 expression and intracellular IFNγ and TNFα production in response to E.G7-OVA cells, was assessed after 3 days. Treatment with Vidu/αQβ enhanced surface expression of CD25 (Fig. 1A–C) and PD-1 (Fig. 1D–F) based on the frequency of positive OT-1 CD8^+^ T cells and median fluorescent intensity (MdFI). Similar expression patterns were seen with SIINFEKL-activated OT-1 CD8^+^ T cells following treatment with Vidu/αQβ, although changes in PD-1 did not reach statistical significance due to high expression by all cells in response to SIINFEKL stimulation (Supplementary Fig. S2).

Treatment with Vidu/αQβ increased intracellular expression of IFNγ (Fig. 1G–I) and TNFα (Fig. 1J–L) by OT-1 CD8^+^ T cells. Another characteristic of CD8^+^ T-cell activation is the production of multiple inflammatory cytokines (e.g., IFNγ and TNFα), referred to as “polyfunctionality” (25). Vidu/αQβ enhanced OT-1 CD8^+^ T-cell polyfunctionality when the cells were stimulated with target E.G7-OVA tumor cells (Fig. 1M and N).

### Vidu decreases proliferation of tumor-specific CD8^+^ T cells while enhancing activation of proliferating cells

**Figure 2.**
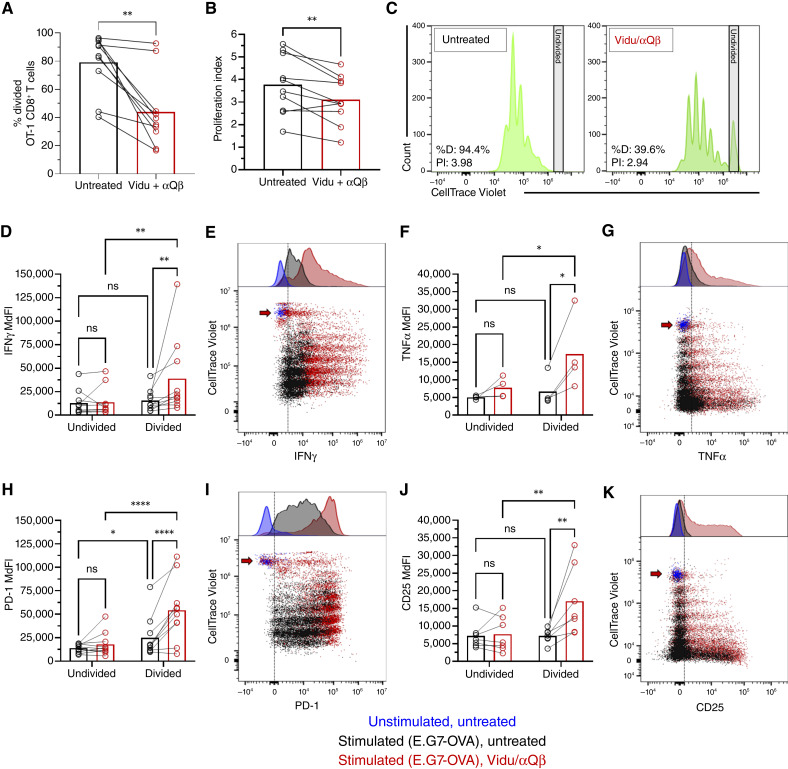
Vidu decreases proliferation of tumor-specific CD8^+^ T cells while enhancing activation of cells that divided. OT-1 splenocytes were labeled with a proliferation tracking dye (CellTrace Violet) and cultured with either media (unstimulated) or E.G7-OVA cells (stimulated) for 1 hour, followed by no additional treatment (untreated) or the addition of Vidu and αQβ (treated). After 3 days of coculture, proliferation and activation marker expression by OT-1 CD8^+^ T cells was analyzed by multicolor spectral flow cytometry. **A,** Percent divided and (**B**) proliferation index of OT-1 CD8^+^ T cells (*n* = 10 mice/group). **C,** Representative histograms showing OT-1 CD8^+^ T-cell proliferation. **D,** MdFI of IFNγ expression by undivided and divided OT-1 CD8^+^ T cells (*n* = 10 mice/group) and (**E**) representative dot plot and histogram in unstimulated (blue), stimulated/untreated (black), and stimulated/treated (red) samples. **F,** MdFI of TNFα expression by undivided and divided OT-1 CD8^+^ T cells (*n* = 4 mice/group) and (**G**) representative dot plot and histogram. **H,** MdFI of PD-1 expression by undivided and divided OT-1 CD8^+^ T cells (*n* = 10 mice/group) and (**I**) representative dot plot and histogram. **J,** MdFI of CD25 expression by undivided and divided OT-1 CD8^+^ T cells (*n* = 7 mice/group) and (**K**) representative dot plot and histogram. **E**, **G**, **I**, and **K,** Red arrows indicate undivided population, and vertical dashed lines indicate separation of negative (left) from positive (right) marker expression. Each symbol connected by a line represents an individual mouse in untreated and Vidu/αQβ conditions and the mean of the group, with error bars indicating the SEM. Statistical significance was determined using a paired *t* test (**A** and **B**) or two-way ANOVA with Sidak multiple comparisons test (**D–J**): *, *P* < 0.05; **, *P* < 0.01; ****, *P* < 0.0001; ns, not significant.

To assess the impact of Vidu/αQβ treatment on the proliferation of tumor-specific CD8^+^ T cells, unfractionated OT-1 splenocytes were labeled with a proliferation dye and cocultured with E.G7-OVA tumor cells followed by the addition of Vidu/αQβ 1 hour later. The frequency of divided OT-1 CD8^+^ T cells and the proliferation index (average number of divisions of cells that do divide) were determined after 3 days. Surprisingly, treatment with Vidu/αQβ decreased both the frequency of dividing OT-1 CD8^+^ T cells (Fig. 2A) and the OT-1 CD8^+^ T-cell proliferation index (Fig. 2B and C). OT-1 CD8^+^ T cells stimulated with SIINFEKL had 100% proliferation with and without Vidu/αQβ treatment, with no differences in the proliferation index (Supplementary Fig. S3A and S3B). The addition of Vidu/αQβ did not affect the proliferation of E.G7-OVA or EL4 tumor cells after 24 hours (Supplementary Fig. S4A–S4D).

Given that Vidu/αQβ treatment increased activation markers expressed by OT-1 CD8^+^ T cells but decreased their proliferation when cultured with antigen-expressing tumor cells, we determined how these two aspects of OT-1 CD8^+^ T-cell activation correlated. Vidu/αQβ treatment increased intracellular expression of IFNγ (Fig. 2D and E) and TNFα (Fig. 2F and G) as well as surface expression of PD-1 (Fig. 2H and I) and CD25 (Fig. 2J and K) by divided OT-1 CD8^+^ T cells but not by undivided cells. Thus, Vidu/αQβ decreased proliferation but increased the activation of divided OT-1 CD8^+^ T cells exposed to target cells.

### Vidu modestly increases granzyme B expression by tumor-specific CD8^+^ T cells but has limited impact on cytotoxicity

**Figure 3.**
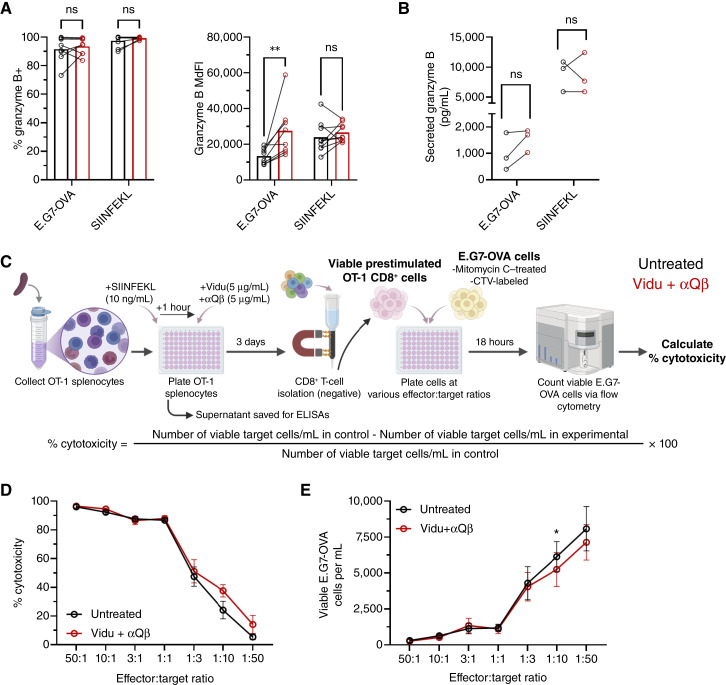
Vidu modestly increases granzyme B expression by tumor-specific CD8^+^ T cells but has limited impact on cytotoxicity. OT-1 splenocytes were cultured with E.G7-OVA cells or SIINFEKL peptide (stimulated) for 1 hour, followed by no additional treatment (untreated) or the addition of Vidu and αQβ (treated). After 3 days of coculture, granzyme B expression or cytotoxicity by OT-1 CD8^+^ T cells was analyzed. **A,** Frequency of granzyme B^+^ OT-1 CD8^+^ T cells and MdFI of granzyme B expression (*n* = 5 mice/group) in untreated (black) and treated (red) samples. **B,** Secreted granzyme B as determined by ELISA (*n* = 3 mice/group). **C,** treatment and cytotoxicity schema of (**D** and **E**) isolated CD8^+^ T cells were cocultured with target E.G7-OVA tumor cells at various effector:target ratios for 18 hours. **D,** Percent cytotoxicity of OT-1 CD8^+^ T cells and (**E**) viable E.G7-OVA cells/mL was determined (*n* = 3–4 mice/group). SIINFEKL peptide was used at a final concentration of 10 ng/mL. Each symbol connected by a line represents an individual mouse in untreated and Vidu/αQβ conditions and the mean of the group, with error bars indicating the SEM. Statistical significance was determined using a paired *t* test: *, *P* < 0.05; **, *P* < 0.01; ns, not significant. [**C,** Created in BioRender. Weiner, G. (2026) https://BioRender.com/s3vjcvy.]

To determine granzyme B expression, OT-1 splenocytes were stimulated with E.G7-OVA coculture or SIINFEKL peptide followed by the addition of Vidu/αQβ 1 hour later. Vidu/αQβ had no impact on the frequency of granzyme B^+^ OT-1 CD8^+^ T cells after 3 days, which was high in all samples (Fig. 3A). Vidu/αQβ treatment significantly increased expression of granzyme B (based on MdFI) by OT-1 CD8^+^ T cells when stimulated with E.G7-OVA cells but not SIINFEKL peptide (Fig. 3A). Vidu/αQβ treatment did not affect the secretion of granzyme B by OT-1 CD8^+^ T cells when stimulated with E.G7-OVA cells or SIINFEKL peptide (Fig. 3B).

To assess how Vidu/αQβ treatment affected the cytotoxic potential of tumor-specific T cells, unfractionated OT-1 splenocytes were stimulated with SIINFEKL peptide followed by the addition of Vidu/αQβ 1 hour later. After 3 days of culture, CD8^+^ T cells were isolated and cytotoxic capacity was evaluated. Isolated CD8^+^ T cells, untreated and treated with Vidu/αQβ, were cocultured with E.G7-OVA tumor cells at various effector:target ratios. The number of target cells (E.G7-OVA) remained fixed, whereas the number of effector cells (OT-1 CD8^+^ T cells) was varied. Target cells were pretreated with mitomycin C (1 μg/mL) to prevent proliferation over the course of the cytotoxicity assay. Effector and target cells were incubated together for 18 hours. After 18 hours, cells were collected, and the absolute count of viable target cells was determined. The experimental setup is summarized in Fig. 3C.

Antitumor activity at higher effector:target ratios (50:1, 10:1, and 3:1) was robust regardless of treatment with Vidu/αQβ. At lower effector:target ratios (1:1, 1:3, 1:10, and 1:50), Vidu/αQβ had a limited impact on the cytotoxicity of tumor-specific CD8^+^ T cells (Fig. 3D and E).

### Vidu enhances triple-positive tumor-specific CD8^+^ T-cell activation marker expression after culture with target cells for 3 days

As reviewed in the introduction, the nomenclature used to describe T-cell exhaustion continues to evolve. For clarity’s sake, cells with a phenotype consistent with progenitor exhaustion (PD-1^+^ and LAG3^+^ but TIM3^−^) will be referred to as “double-positive,” and those with a phenotype consistent with terminal exhaustion (PD-1^+^, LAG3^+^, and TIM3^+^) will be referred to as “triple-positive.” This is done to avoid confusing phenotypic expression of inhibitory receptors with the functionality of various exhausted T-cell populations.

**Figure 4.**
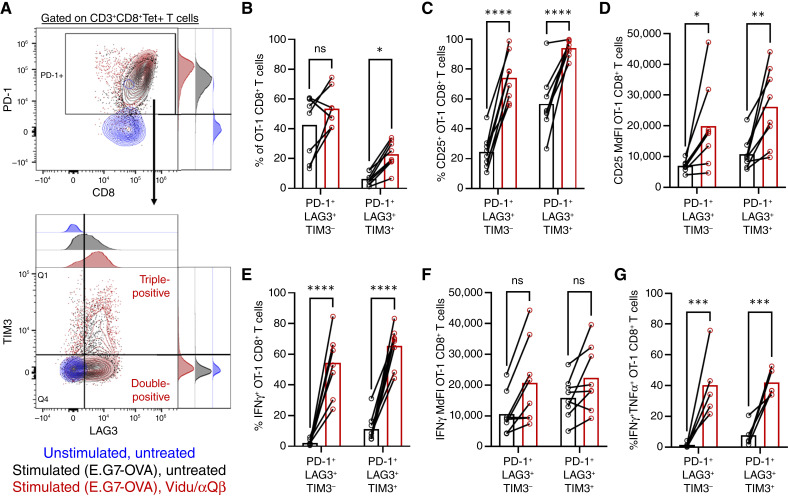
Vidu enhances triple-positive tumor-specific CD8^+^ T-cell activation marker expression after culture with target cells for 3 days. OT-1 splenocytes were cultured with either media (unstimulated) or E.G7-OVA cells (stimulated) for 1 hour, followed by no additional treatment (untreated) or the addition of Vidu and αQβ (treated). After 3 days of coculture, activation and exhaustion marker expression by OT-1 CD8^+^ T cells was analyzed by multicolor spectral flow cytometry. **A,** Representative dot plot and histogram in unstimulated (blue), stimulated/untreated (black), and stimulated/treated (red) samples showing the gating strategy of PD-1^+^ OT-1 CD8^+^ T cells and their coexpression of LAG3 ± TIM3. **B,** Double- and triple-positive OT-1 CD8^+^ T-cell populations were analyzed for activation marker expression in untreated (black) and treated (red) samples (*n* = 8 mice/group). **C,** Frequency of CD25^+^ OT-1 CD8^+^ T cells and (**D**) MdFI of CD25 expression (*n* = 8 mice/group). **E,** Frequency of IFNγ^+^ OT-1 CD8^+^ T cells and (**F**) MdFI of IFNγ expression (*n* = 8 mice/group). **G,** Frequency of IFNγ^+^TNFα^+^ OT-1 CD8^+^ T cells (*n* = 5 mice/group). Each symbol connected by a line represents an individual mouse in untreated and Vidu/αQβ conditions and the mean of the group, with error bars indicating the SEM. Statistical significance was determined using a two-way ANOVA with Sidak multiple comparisons test: *, *P* < 0.05; **, *P* < 0.01; ***, *P* < 0.001; ****, *P* < 0.0001; ns, not significant.

Unfractionated OT-1 splenocytes were stimulated with E.G7-OVA tumor cells for 1 hour followed by the addition of Vidu/αQβ and cultured for 3 days. Coexpression of PD-1, LAG3, and TIM3 was then determined (Fig. 4A). Whereas treatment with Vidu/αQβ had no significant impact on the fraction of OT-1 CD8^+^ T cells that were double-positive, the addition of Vidu/αQβ significantly increased the frequency of CD8^+^ T cells that were triple-positive (Fig. 4B). Despite increasing the frequency of triple-positive cells, Vidu/αQβ treatment enhanced expression of markers associated with activation, including surface CD25 (Fig. 4C and D) and intracellular IFNγ (Fig. 4E and F) in both double- and triple-positive populations. Vidu/αQβ treatment also increased the frequency of polyfunctional OT-1 CD8^+^ T cells in both the double- and triple-positive populations compared with untreated controls (Fig. 4G).

Thus, Vidu/αQβ increased the frequency of triple-positive OT-1 CD8^+^ T cells after 3 days. Despite appearing more “exhausted” based on surface inhibitory marker coexpression, Vidu/αQβ-treated triple-positive OT-1 CD8^+^ T cells had increased expression of activation markers.

### Vidu has complex effects on the expression of markers of activation and exhaustion after long-term *in vitro* culture

**Figure 5.**
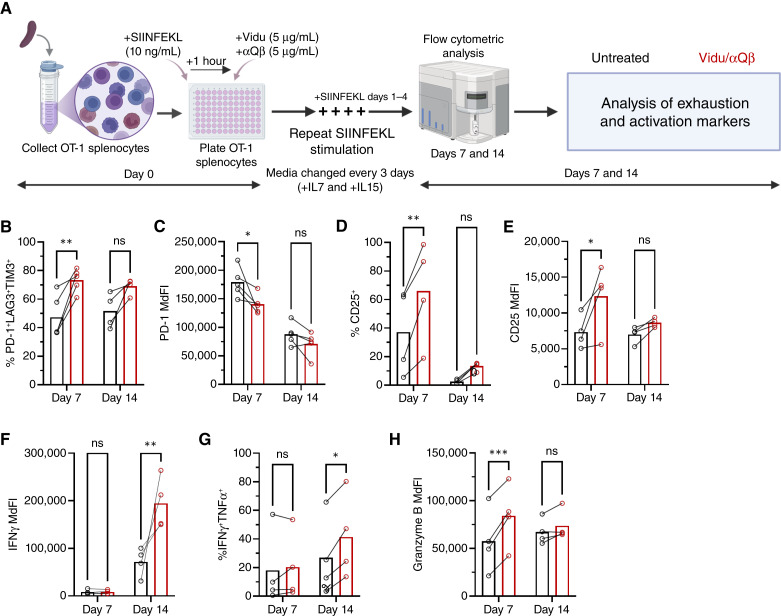
Vidu has complex effects on the expression of markers of activation and exhaustion after long-term *in vitro* culture. **A,** OT-1 splenocytes were cultured with SIINFEKL peptide (stimulated) for 1 hour, followed by no additional treatment (untreated) or the addition of Vidu and αQβ (treated). Additional SIINFEKL stimulation on days 1–4 for a total of 5 SIINFEKL doses was provided to evaluate chronic antigen stimulation on OT-1 CD8^+^ T cells. After 7 and 14 days of culture, activation and exhaustion marker expression by OT-1 CD8^+^ T cells was analyzed by multicolor spectral flow cytometry. Media were changed every 3 days with supplemental IL7 and IL15. Exhaustion status of OT-1 CD8^+^ T cells was determined by the (**B**) frequency of triple-positive OT-1 CD8^+^ T cells and (**C**) MdFI of PD-1 on OT-1 CD8^+^ T cells (*n* = 4–5 mice/group) in untreated (black) and treated (red) samples. Activation status of OT-1 CD8^+^ T cells was determined by the (**D**) frequency of CD25^+^ OT-1 CD8^+^ T cells and (**E**) MdFI of CD25 expression (*n* = 4 mice/group). CD8^+^ (**F**) MdFI of IFNγ, (**G**) frequency of IFNγ^+^TNFα^+^, and (**H**) MdFI of granzyme B expressed by OT-1 CD8^+^ T cells (*n* = 4 mice/group). Each symbol connected by a line represents an individual mouse in untreated and Vidu/αQβ conditions and the mean of the group, with error bars indicating the SEM. Statistical significance was determined using a two-way ANOVA with Sidak multiple comparisons test: *, *P* < 0.05; **, *P* < 0.01; ***, *P* < 0.001; ns, not significant. [**A,** Created in BioRender. Weiner, G. (2026) https://BioRender.com/s3vjcvy.]

It is well-established that the induction of T-cell exhaustion requires prolonged antigen exposure from target cells to T cells (26–28). To assess the impact of Vidu/αQβ on tumor-specific CD8^+^ T cells over time, an *in vitro* stimulation assay was utilized to assess chronic antigen exposure to OT-1 CD8^+^ T cells (Fig. 5A; ref. 29). SIINFEKL stimulation was chosen for long-term cultures as E.G7-OVA tumor cells overgrew OT-1 cells after 3 days. Unfractionated OT-1 splenocytes were stimulated with SIINFEKL peptide for 1 hour, followed by the addition of Vidu/αQβ. Additional SIINFEKL was added daily from days 1 to 4 to mimic chronic antigen stimulation. Cells were analyzed on days 7 and 14.

Vidu/αQβ significantly increased the proportion of triple-positive OT-1 CD8^+^ T cells on day 7, with a modest increase persisting on day 14 (Fig. 5B). Although all OT-1 CD8^+^ T cells expressed PD-1 on days 7 and 14 (Supplementary Fig. S5), Vidu/αQβ treatment resulted in lower expression of PD-1 (based on MdFI) on day 7. This difference was no longer significant by day 14 (Fig. 5C). Treatment with Vidu/αQβ increased surface expression of CD25 on day 7. As with expression of PD-1, differences in CD25 expression were no longer significant by day 14 (Fig. 5D and E). Vidu/αQβ-treated OT-1 CD8^+^ T cells restimulated with SIINFEKL 24 hours before analysis did not have increased intracellular IFNγ on day 7 but did on day 14 (Fig. 5F). Vidu/αQβ also increased OT-1 CD8^+^ T-cell polyfunctionality, as indicated by coexpression of IFNγ and TNFα, on day 14 (Fig. 5G). The granzyme B recall response of OT-1 CD8^+^ T cells was elevated with Vidu/αQβ treatment on day 7 but not on day 14 (Fig. 5H).

Overall, Vidu/αQβ treatment with chronic antigen stimulation modestly increased the number of tumor-specific T cells with a triple-positive phenotype. These cells retained markers of activation, polyfunctionality, and cytotoxic molecule expression. The challenges with maintaining longer term cultures also speak to the importance of evaluating the response to Vidu on tumor-specific T cells *in vivo*.

### IT Vidu injections expand tumor-specific CD8^+^ T cells in the tumor and enhance antitumor activity

**Figure 6.**
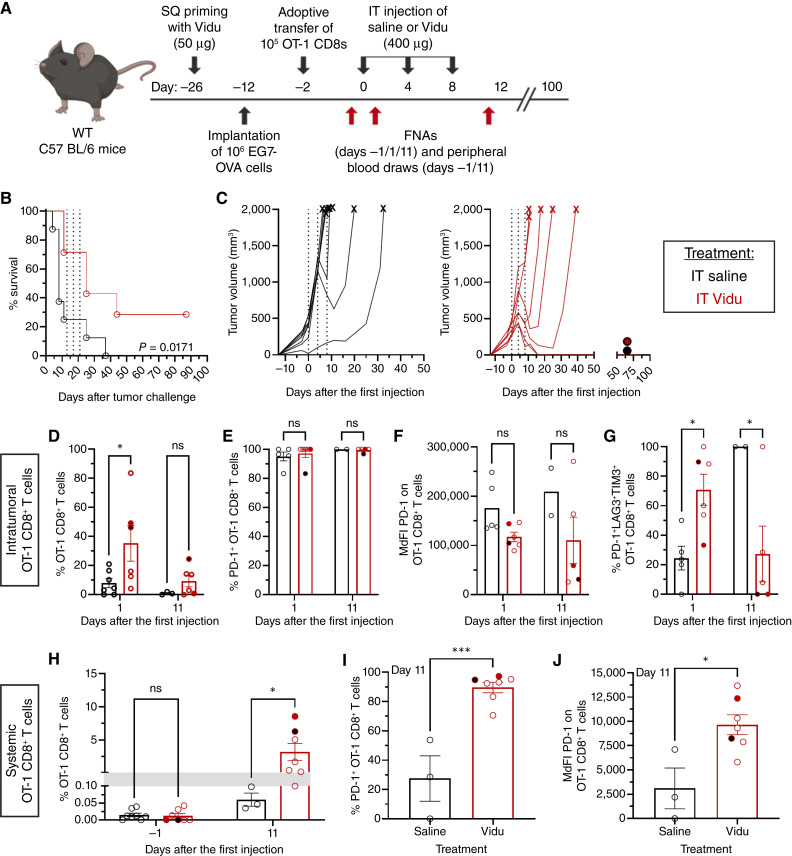
IT Vidu injections enhance tumor regression and markedly increase the number of circulating tumor-specific T cells. **A,***In vivo* treatment schema evaluating the impact of IT Vidu treatment. CD8^+^CD8^+^. **B,** Kaplan–Meyer curve of saline (untreated, black) vs. Vidu (treated, red) mouse survival (*n* = 7–8 mice/group). **C,** Spider plots of saline (left) vs. Vidu (right) treated mice showing individual tumor volumes over time; dashed vertical lines indicate day of IT injection, “X” indicates the time point an individual mouse was sacrificed, and a filled circle indicates a mouse surviving to the endpoint of the study (*n* = 7–8 mice/group). **D,** Frequency of IT tumor-specific CD8^+^ T cells, as indicated by tetramer positivity, out of total CD8^+^ T cells (*n* = 3–7 mice/group). **E,** Frequency of IT PD-1^+^ OT-1 CD8^+^ T cells and (**F**) MdFI of PD-1 expression (*n* = 2–6 mice/group). **G,** Frequency of IT PD-1^+^LAG3^+^TIM3^+^ tumor-specific CD8^+^ T cells (*n* = 2–6 mice/group). **H,** Frequency of circulating tumor-specific CD8^+^ T cells out of total CD8^+^ T cells (*n* = 3–8 mice/group). **I,** Frequency of circulating PD-1^+^ OT-1 CD8^+^ T cells and (**J**) MdFI of PD-1 expression (*n* = 3–7 mice/group). Each symbol represents an individual mouse and the mean of the group, with error bars indicating the SD. Statistical significance was determined using a (**B**) log-rank (Mantel–Cox) test for survival, (**D–H**) two-way ANOVA with Sidak multiple comparisons test and (**I** and **J**) unpaired *t* test: *, *P* < 0.05; ***, *P* < 0.001; ns, not significant. [**A,** Created in BioRender. Weiner, G. (2026) https://BioRender.com/s3vjcvy.]

An *in vivo* study design, illustrated in Fig. 6A, was used to assess the impact of Vidu therapy on tumor-specific CD8^+^ T cells under conditions where the number of tumor-specific T cells was insufficient to induce spontaneous tumor regression without treatment. Thus, tumors were established before a relatively small number of OT-1 cells were transferred (see “Materials and Methods”). This allowed for longitudinal tracking of IT tumor-specific T cells while limiting robust and rapid tumor regression that would result from the transfer of larger numbers of such cells earlier in tumor development.

Under these conditions, tumor growth was robust in mice treated IT with saline. IT Vidu had a modest impact on survival (Fig. 6B) and tumor burden (Fig. 6C) compared with saline controls. Some mice experienced tumor regrowth after Vidu treatment was stopped, whereas two Vidu-treated mice remained tumor-free. Pretreatment FNAs and blood samples revealed minimal OT-1 CD8^+^ T cells. No significant differences between the Vidu and saline groups were observed in the number of overall CD8^+^ T cells in either the tumor or circulation (Supplementary Fig. S6).

On day 1 after the first IT injection, the proportion of OT-1 CD8^+^ T cells within the tumor was higher in Vidu-treated compared with saline-treated mice (Fig. 6D). This trend continued but was no longer statistically significant on day 11 (Fig. 6D). Nearly all OT-1 CD8^+^ T cells in the tumor expressed PD-1 on days 1 and 11 (Fig. 6E). PD-1 expression levels, measured by MdFI, were lower in Vidu-treated mice on day 1 and slightly reduced on day 11 compared with saline-treated mice (Fig. 6F). On day 1, IT OT-1 CD8^+^ T cells in Vidu-treated mice exhibited a greater proportion of triple positive cells compared with saline-treated mice. This pattern reversed on day 11 when the frequency of triple-positive cells was lower in Vidu-treated mice compared with saline-treated mice (Fig. 6G). The two Vidu-treated mice with no detectable triple positive OT-1 CD8^+^ T cells at this time point were the only animals to survive to the study endpoint.

These findings demonstrate a transient effect of Vidu treatment on activation and exhaustion phenotypes of IT tumor-specific T cells which is largely consistent with observations made *in vitro*.

### IT Vidu injections enhanced the number of circulating tumor-specific CD8^+^ T cells

Circulating tumor-specific CD8^+^ T cells on day 11 were more than 50-fold higher in Vidu-treated mice compared with saline-treated mice (Fig. 6H). Circulating tumor-specific CD8^+^ T cells in Vidu-treated mice had higher PD-1 expression than those from saline-treated mice (Fig. 6I and J). Unlike their IT counterparts (Fig. 6G), PD-1–positive circulating OT-1 CD8^+^ T cells did not coexpress LAG3 and TIM3.

Collectively, these findings indicate that IT Vidu treatment expands tumor-specific CD8^+^ T cells both within the tumor and in circulation, with the most pronounced increase observed in the circulation. Whereas tumor-resident tumor-specific T cells had phenotypic features of exhaustion, their circulating counterparts did not.

### αPD-1 enhanced the antitumor activity of Vidu and sustained IT T cells

**Figure 7.**
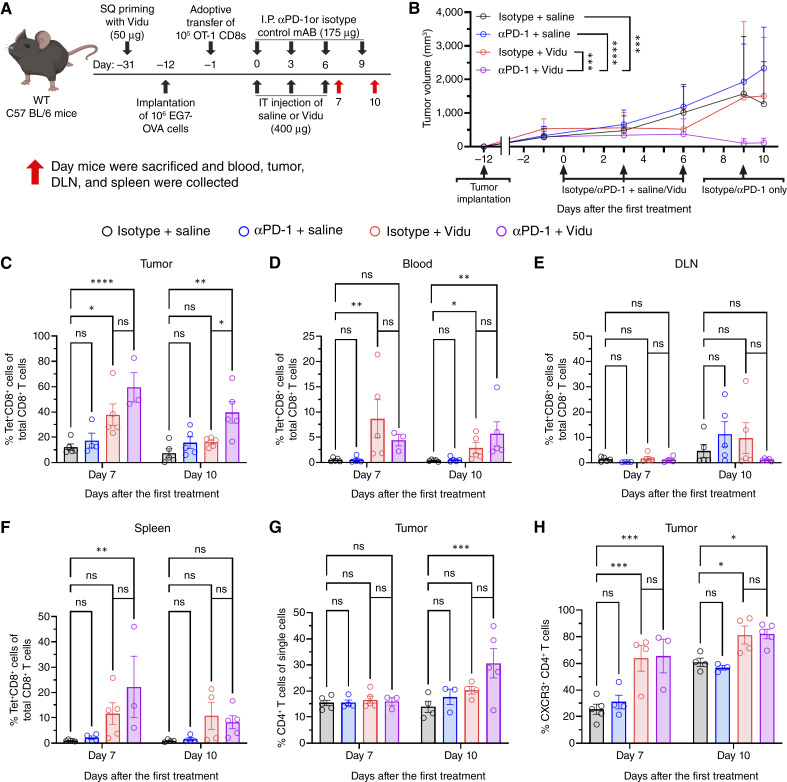
αPD-1 enhanced the antitumor activity of Vidu and sustained IT T cells. **A,***In vivo* treatment schema evaluating the impact of combination αPD-1 and Vidu CD8^+^CD8^+^. **B,** Tumor volumes over time; arrows indicate day of tumor implantation and treatment (*n* = 4–10 mice/group). **C,** Frequency of IT tumor-specific CD8^+^ T cells, as indicated by tetramer positivity, out of total CD8^+^ T cells. Frequency of tumor-specific CD8^+^ T cells out of total CD8^+^ T cells in (**D**) circulation, (**E**) DLN, and (**F**) spleen. Frequency of IT (**G**) CD4^+^ T cells out of single cells. **H,** Frequency of IT CXCR3^+^ CD4^+^ T cells out of total CD4^+^ T cells (*n* = 3–5 mice/group). Each symbol represents an individual mouse and the mean of the group with SD. Statistical significance was determined using a (**B–H**) one-way ANOVA with Tukey test for multiple comparisons and (**C–H**) unpaired *t* test between Vidu and combination groups: *, *P* < 0.05; **, *P* < 0.01; ***, *P* < 0.001; ****, *P* < 0.0001; ns, not significant. [**A,** Created in BioRender. Weiner, G. (2026) https://BioRender.com/s3vjcvy.]

The therapeutic regimen for *in vivo* studies, illustrated in Fig. 7A, was designed to study the impact of Vidu with and without αPD-1 therapy on tumor-specific CD8^+^ T cells. Mice were sacrificed on day 7 or day 10 to assess immune cell populations 24 hours and 4 days after the cessation of IT Vidu therapy. Cells from blood, tumors, DLNs, and spleens were analyzed by flow cytometry.

Vidu, with and without αPD-1, slowed tumor growth through day 7. Those mice treated with single-agent IT Vidu had tumor regrowth after Vidu was stopped. Mice treated with combination therapy had sustained tumor control, with multiple mice achieving tumor-free status by day 10 (Fig. 7B). Vidu-treated mice, with and without αPD-1, had an increased percentage of tumor-specific CD8^+^ T cells in the tumor on day 7 compared with isotype/saline-treated mice. On day 10, only mice treated with combination therapy had a significantly higher frequency of tumor-specific CD8^+^ T cells in the tumor (Fig. 7C; Supplementary Fig. S7A). Together, these data suggest that antitumor T-cell numbers and activity induced by Vidu is sustained by αPD-1 therapy.

The addition of αPD-1 to Vidu treatment did not significantly alter the frequency of tumor-specific CD8^+^ T cells in circulation on day 7 or day 10 (Fig. 7D). Treatment had no impact on the frequency of tumor-specific CD8^+^ T cells in the DLNs on day 7 or day 10 (Fig. 7E). Mice treated with combination therapy had an increase in the frequency of tumor-specific CD8^+^ T cells in the spleen on day 7 but not day 10 (Fig. 7F).

Mice treated with combination therapy had an increased frequency of total CD4^+^ T cells in the tumor on day 10 compared with isotype/saline-treated mice (Fig. 7G). Treatment had no impact on the total number of CD4^+^ T cells in the tumor on day 7 or day 10 (Supplementary Fig. S7B). Vidu, with and without αPD-1, increased the frequency of CXCR3^+^ CD4^+^ T cells in the tumor on both day 7 and day 10 (Fig. 7H).

## Discussion

The goal of *in situ* immunization is to modify the TME to enhance the local and systemic antitumor T-cell response. *In situ* immunization with Vidu has demonstrated clinical activity, particularly when combined with αPD-1 therapy (15). Responses include regression of noninjected and distant tumors. Despite these promising results, many patients do not respond to therapy or respond only transiently. The impact of *in situ* immunization in general and *in situ* immunization with Vidu in particular on tumor-specific T-cell proliferation, activation, and exhaustion is not fully understood. In this study, we utilized the well-established OT-1 mouse model to investigate how Vidu alters the tumor-specific CD8^+^ T-cell response *in vitro* and *in vivo*.

*In vitro*, Vidu affected tumor-specific T-cell proliferation, activation, and expression of exhaustion markers in ways that illustrate the complexity of the relationship between T-cell phenotype and function. Surprisingly, Vidu reduced proliferation of tumor-stimulated CD8^+^ T cells while increasing expression of activation markers, such as CD25 and IFNγ, in those CD8^+^ cells that did proliferate. Vidu-treated CD8^+^ T cells maintained a robust activation phenotype and polyfunctional capabilities after chronic stimulation despite many cells being triple-positive (PD-1^+^LAG3^+^TIM3^+^) and thus appearing more “terminally exhausted.” Vidu did not have a major impact on the cytotoxicity of tumor-specific CD8^+^ T cells *in vitro*. These findings suggest that Vidu can increase the phenotypic appearance of exhaustion while also preserving activation and cytotoxic potential.

The minimal impact of Vidu on short-term *in vitro* cytotoxicity does not fully explain robust antitumor response seen *in vivo*. Indeed, IT Vidu treatment increased IT tumor-specific CD8^+^ T cells and suppressed tumor growth during treatment. However, tumors regrew rapidly in mice treated with single-agent IT Vidu after Vidu therapy was stopped. In contrast, mice treated with both Vidu and αPD-1 had sustained tumor control for the remainder of the experiment. Combination therapy also correlated with a sustained increase in IT tumor-specific CD8^+^ T cells compared with untreated mice or those treated with Vidu alone.

IT Vidu increased tumor-specific CD8^+^ T cells found in circulation. These cells did not have an exhausted phenotype. The addition of αPD-1 did not have a significant impact on the circulating tumor-specific T-cell population in contrast to the effect αPD-1 had on IT tumor-specific T cells. This suggests that Vidu promotes systemic antitumor immunity by enhancing the export of nonexhausted tumor-specific T cells whereas the addition of αPD-1 enhances the sustenance and functional activity of the tumor-specific T cells within the TME. These results provide further mechanistic rationale for combining Vidu with αPD-1. Indeed, our prior studies in mice, and clinical trials demonstrate that IT Vidu can induce regression of both injected and noninjected lesions, particularly when used in combination with αPD-1 (9, 30). Our laboratory has previously demonstrated that the antitumor activity of Vidu is dependent on both CD4^+^ and CD8^+^ T cells (9). Here, we confirm that Vidu therapy increases Th1 CD4^+^ T cells in the tumor and could contribute to the antitumor CD8^+^ T-cell response although the specificity of the CD4^+^ T-cell response was not specifically defined (31).

As with all models, the studies reported here have limitations. The expression pattern of TLR9 in humans and mice varies, with expression being limited to B cells and pDCs in humans and more broadly expressed in B cells, monocytes, macrophages, and DCs in mice (8, 32, 33). Despite this varied TLR9 expression between humans and mice, there are similarities in the antitumor T-cell response induced by IT Vidu in both systems. The OT-1 model is not designed to explore the role played by tumor-specific CD4^+^ T cells. Despite these limitations, the studies reported here provide important insight into the impact of *in situ* immunization with Vidu, either alone or combined with αPD-1, on tumor-specific T cells. Implications of our findings could affect clinical development of related strategies designed to alter the TME to enhance the antitumor immune response.

## Supplementary Material

Supplementary Figure S1 Figure S1. Flow cytometry gating strategy to identify OT-1 CD8+ T cells and their activation and inhibitory marker expression.

Supplementary Figure S2 Figure S2. Addition of SIINFEKL peptide followed by Vidu/αQβ treatment increases OT-1 CD8+ T cell activation marker expression.

Supplementary Figure S3 Figure S3. Proliferation of OT-1 CD8+ T cells when stimulated with SIINFEKL peptide.

Supplementary Figure S4 Figure S4. Proliferation of EL4 or E.G7-OVA tumor cell proliferation.

Supplementary Figure S5 Supplementary Figure S5. Treatment with Vidu/αQβ alters activation and inhibitory marker expression by OT-1 CD8+ T cells that received a single or repeat SIINFEKL doses.

Supplementary Figure S6 Figure S6. The number of overall CD8+ T cells overall in the tumor and in the blood does not change over the course of Vidu treatment.

Supplementary Figure S7 Figure S7. The number of tumor-specific CD8+ T cells but not the number of total CD4+ T cells per gram of tumor increases with Vidu therapy.

## Data Availability

The data generated in this study are available upon request to the corresponding author.
