# Supplementary material for: Intratumoral Virus-Like Particles Containing a TLR9 Agonist Combined with Systemic αPD-1 Activate Tumor-Specific CD8+ T Cells
Source: Cancer Res Commun. 2026 May 1;6(5):1006–19. doi: 10.1158/2767-9764.CRC-26-0175 (PMC13133427; doi:10.1158/2767-9764.CRC-26-0175)
Supplement: Supplementary Figure S1 — Figure S1. Flow cytometry gating strategy to identify OT-1 CD8+ T cells and their activation and inhibitory marker expression. [file crc-26-0175_supplementary_figure_s1_suppsf1.pdf]

Supplemental Figure 1

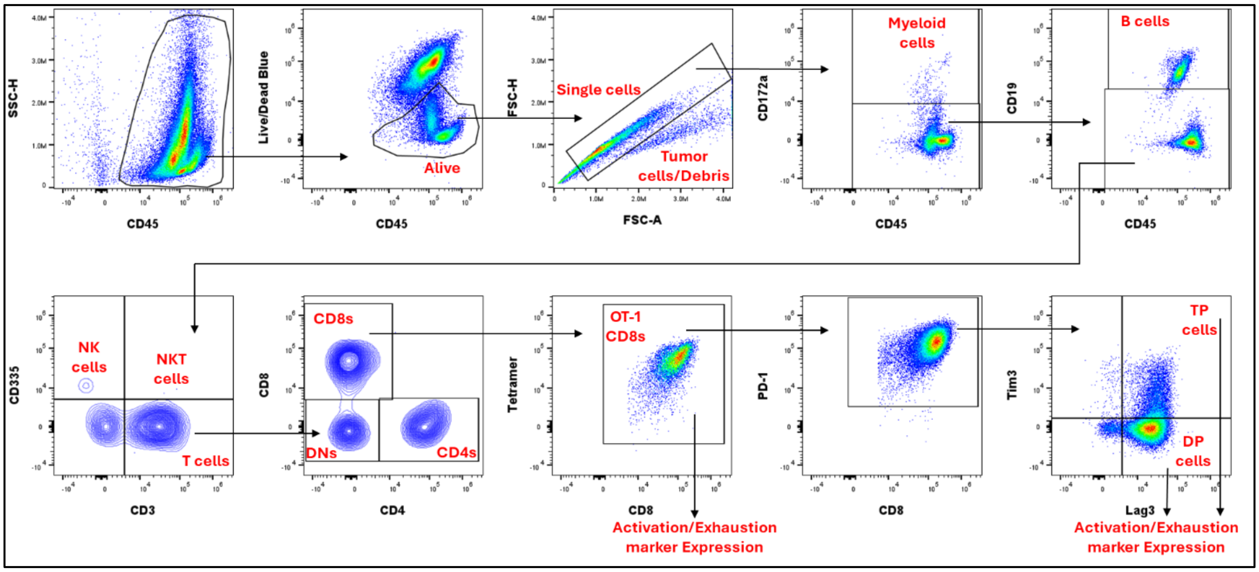

Supplemental Figure 1 Flow cytometry gating strategy to identify OT-1 CD8^{+} T cells and their activation and inhibitory marker expression. Splenocytes from one OT-1 mouse were stained with immune cell markers and acquired on the Cytek Aurora. Gating strategy was done using Flowjo software. OT-1 CD8^{+} T cells were gated to be: (1) CD45+, (2) Live/Dead Blue negative/live, (3) single cells, (4) CD172α-, (5) CD19-, (6) CD335-CD3+, (7) CD8+CD4-. OT-1 CD8+ T cells were then identified as Tetramer+ and marker expression was determined.
