# Supplementary material for: Intratumoral Virus-Like Particles Containing a TLR9 Agonist Combined with Systemic αPD-1 Activate Tumor-Specific CD8+ T Cells
Source: Cancer Res Commun. 2026 May 1;6(5):1006–19. doi: 10.1158/2767-9764.CRC-26-0175 (PMC13133427; doi:10.1158/2767-9764.CRC-26-0175)
Supplement: Supplementary Figure S2 — Figure S2. Addition of SIINFEKL peptide followed by Vidu/αQβ treatment increases OT-1 CD8+ T cell activation marker expression. [file crc-26-0175_supplementary_figure_s2_suppsf2.pdf]

Supplemental Figure 2

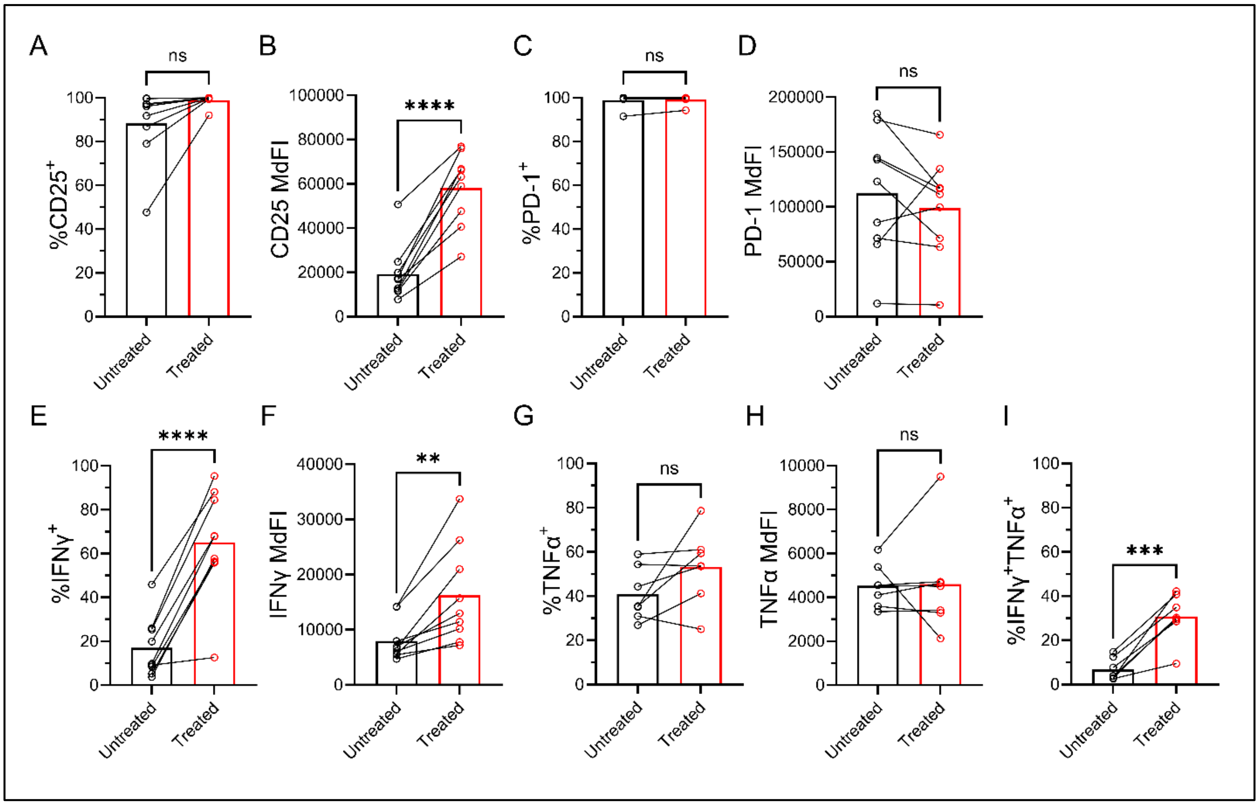

Supplemental Figure 2 Addition of SIINFEKL peptide followed by Vidu/αQβ treatment increases OT-1 $CD8^+$ T cell activation marker expression. (A) Frequency of $CD25^+$ OT-1 $CD8^+$ T cells and (B) MdFI of CD25 expression in untreated (black) and treated (red) samples. (C) Frequency of PD-1^+ OT-1 $CD8^+$ T cells and (D) MdFI of PD-1 expression. (E) Frequency of $IFN\gamma^+$ OT-1 $CD8^+$ T cells and (F) MdFI of IFNγ expression. (G) Frequency of $TNF\alpha^+$ OT-1 $CD8^+$ T cells and (H) MdFI of TNFα expression. (I) Frequency of polyfunctional $IFN\gamma^+TNF\alpha^+$ OT-1 $CD8^+$ T cells. Splenocytes from 7-9 OT-1 mice were plated and stimulated with SIINFEKL peptide (10ng/mL) for 1 hour followed by Vidu/αQβ treatment (5ug/mL each). Cells were analyzed after 3 days by flow cytometry. Data shown is mean ± SEM. Statistical significance was determined using a paired t-test: **p<0.01, ***p<0.001, ****p<0.0001, ns, not significant.
