# Supplementary material for: Intratumoral Virus-Like Particles Containing a TLR9 Agonist Combined with Systemic αPD-1 Activate Tumor-Specific CD8+ T Cells
Source: Cancer Res Commun. 2026 May 1;6(5):1006–19. doi: 10.1158/2767-9764.CRC-26-0175 (PMC13133427; doi:10.1158/2767-9764.CRC-26-0175)
Supplement: Supplementary Figure S3 — Figure S3. Proliferation of OT-1 CD8+ T cells when stimulated with SIINFEKL peptide. [file crc-26-0175_supplementary_figure_s3_suppsf3.pdf]

Supplemental Figure 3

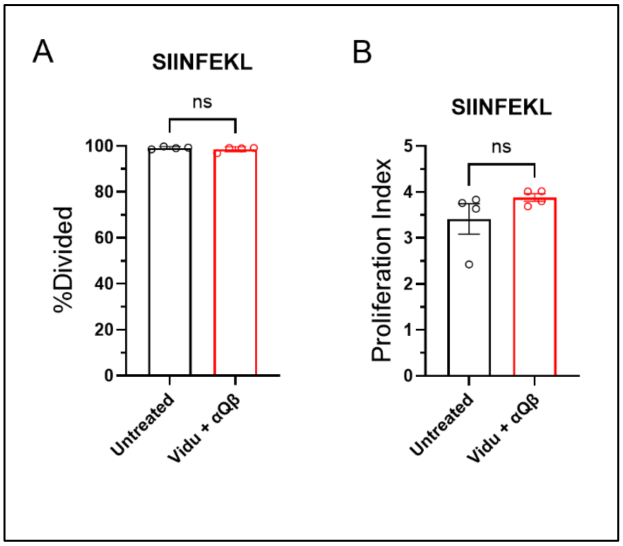

Supplemental Figure 3 Proliferation of OT-1 CD8+ T cells when stimulated with SIINFEKL peptide. OT-1 splenocytes were labeled with a proliferation tracking dye (CellTrace Violet) and cultured with either media (unstimulated) or SIINFEKL peptide (stimulated) for 1 hour, followed by no additional treatment (untreated) or the addition of Vidu and αQβ (treated). After 3 days of co-culture, proliferation and activation marker expression by OT-1 $CD8^{+}$ T cells was analyzed by multicolor spectral flow cytometry. (A) Percent divided and (B) Proliferation Index of OT-1 $CD8^{+}$ T cells (n=4). SIIFNEKL was used at a final concentration of 10ng/mL
