# Supplementary material for: Intratumoral Virus-Like Particles Containing a TLR9 Agonist Combined with Systemic αPD-1 Activate Tumor-Specific CD8+ T Cells
Source: Cancer Res Commun. 2026 May 1;6(5):1006–19. doi: 10.1158/2767-9764.CRC-26-0175 (PMC13133427; doi:10.1158/2767-9764.CRC-26-0175)
Supplement: Supplementary Figure S4 — Figure S4. Proliferation of EL4 or E.G7-OVA tumor cell proliferation. [file crc-26-0175_supplementary_figure_s4_suppsf4.pdf]

Supplemental Figure 4

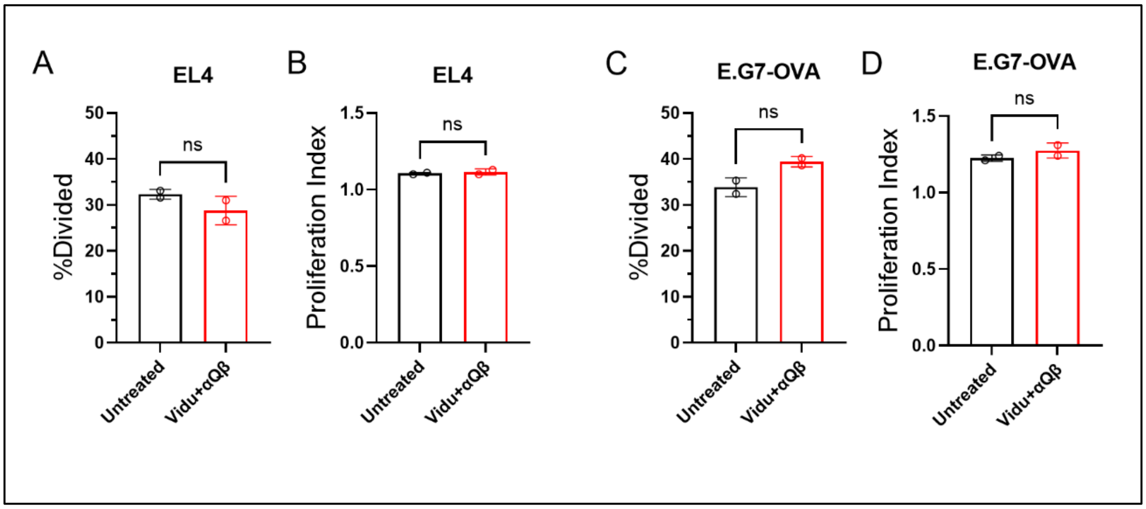

Supplemental Figure 4 Proliferation of EL4 or E.G7-OVA tumor cell proliferation. Tumor cells were labeled with a proliferation tracking dye (CellTrace Violet) and were treated with or without Vidu and αQβ. After 24 hours, proliferation was analyzed by multicolor spectral flow cytometry. EL4 (A) Percent divided and (B) Proliferation Index and E.G7-OVA (C) Percent divided and (D) Proliferation index (n=1). Individual points represent technical replicates. SIINFEKL was used at a final concentration of 10ng/mL (n=1)
