# Supplementary material for: Intratumoral Virus-Like Particles Containing a TLR9 Agonist Combined with Systemic αPD-1 Activate Tumor-Specific CD8+ T Cells
Source: Cancer Res Commun. 2026 May 1;6(5):1006–19. doi: 10.1158/2767-9764.CRC-26-0175 (PMC13133427; doi:10.1158/2767-9764.CRC-26-0175)
Supplement: Supplementary Figure S5 — Treatment with Vidu/αQβ alters activation and inhibitory marker expression by OT-1 CD8+ T cells that received a single or repeat SIINFEKL doses. [file crc-26-0175_supplementary_figure_s5_suppsf5.pdf]

Supplemental Figure 5

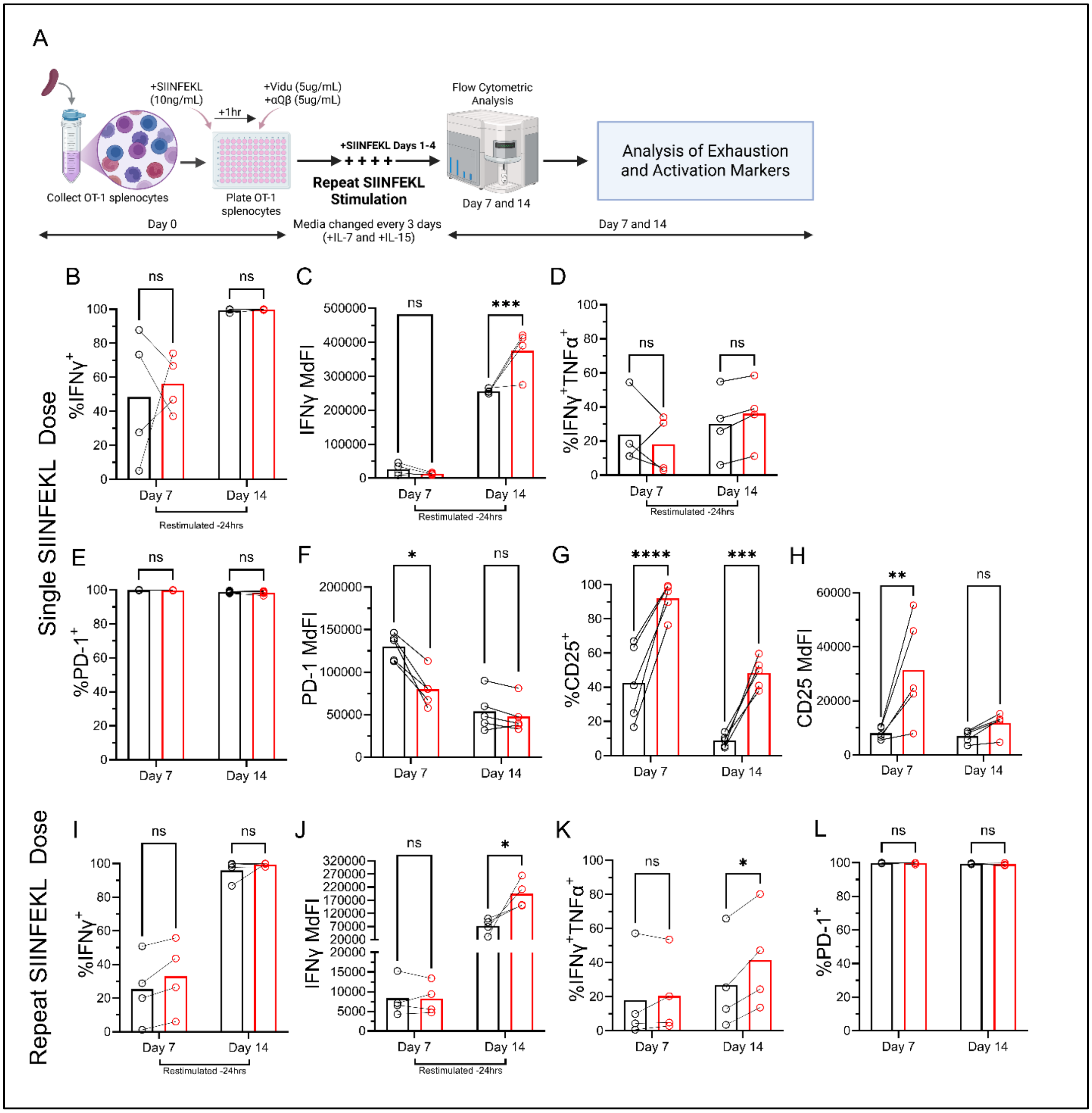

Supplemental Figure 5 Treatment with Vidu/αQβ alters activation and inhibitory marker expression by OT-1 CD8+ T cells that received a single or repeat SIINFEKL doses. Splenocytes from 4-5 OT-1 mice were pre-stimulated with SIINFEKL peptide (10ng/mL) for 1 hour followed by treatment with Vidu/αQβ (5ug/mL each). Two stimulation groups were tested: (B-H) "Single SIINFEKL Dose" group received SIINFEKL at the Day 0 timepoint only, (I-M) "Repeated SIINFEKL Doses" group received SIINFEKL at the Day 0 timepoint with additional SIINFEKL (10ng/mL) added on Days 1 to 4. After 7 and 14 days, marker expression was evaluated via Flow Cytometry. Activation status of Single SIINFEKL Dose

OT-1 CD8$^+$ T cells was determined by (B) frequency of IFNγ$^+$ OT-1 CD8$^+$ T cells and (C) MdFI of IFNγ expression, (D) frequency of IFNγ$^+$TNFα$^+$ OT-1 CD8$^+$ T cells, (E) frequency of PD-1$^+$ OT-1 CD8$^+$ T cells and (F) MdFI of PD-1 expression, (G) frequency of CD25$^+$ OT-1 CD8$^+$ T cells and (H) MdFI of CD25 expression (n=4-5 mice/group). Activation status of Repeat SIINFEKL Dose OT-1 CD8$^+$ T cells was determined by (I) frequency of IFNγ$^+$ OT-1 CD8$^+$ T cells and (J) MdFI of IFNγ expression, (K) frequency of IFNγ$^+$TNFα$^+$ OT-1 CD8$^+$ T cells, (L) frequency of PD-1$^+$ OT-1 CD8$^+$ T cells (n=4-5 mice/group). Statistical significance was determined using a two-way ANOVA with Sidak's multiple comparisons test: *$p<0.05$, **$p<0.01$, ***$p<0.001$, ****$p<0.0001$, ns, not significant. (A) Created with Biorender.com
