# Supplementary material for: Intratumoral Virus-Like Particles Containing a TLR9 Agonist Combined with Systemic αPD-1 Activate Tumor-Specific CD8+ T Cells
Source: Cancer Res Commun. 2026 May 1;6(5):1006–19. doi: 10.1158/2767-9764.CRC-26-0175 (PMC13133427; doi:10.1158/2767-9764.CRC-26-0175)
Supplement: Supplementary Figure S6 — Figure S6. The number of overall CD8+ T cells overall in the tumor and in the blood does not change over the course of Vidu treatment. [file crc-26-0175_supplementary_figure_s6_suppsf6.pdf]

Supplemental Figure 6

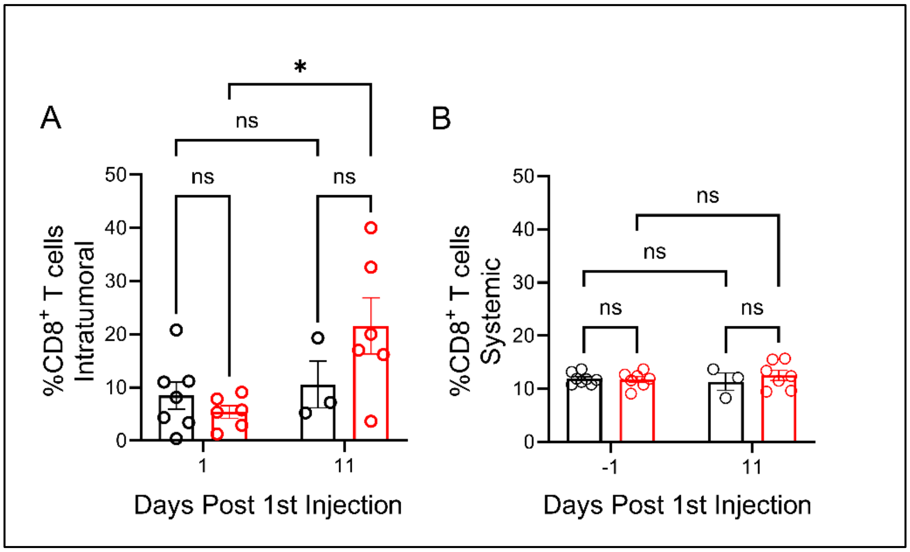

Supplemental Figure 6 The number of overall CD8+ T cells overall in the tumor and in the blood does not change over the course of Vidu treatment. Wild Type C57BL/6 mice were primed with Vidu (Day –26) followed by implantation SQ in the left flank with 10^6 EG7-OVA tumor cells (Day –12) and adoptive transfer of OT-1 splenocytes corresponding to 10^5 OT-1 CD8+ T cells (Day –2). Mice then received intratumoral injections of either saline or Vidu on Days 0, 4, and 8 and T cell population in the tumor and blood were monitored. (A) Percent Intratumoral overall CD8+ T cells out of single cells and (B) Percent systemic overall CD8+ T cells out of single cells. Statistical significance was determined using a two-way ANOVA with Sidak's multiple comparisons test: *p<0.05, ns, not significant.
