# Supplementary material for: Intratumoral Virus-Like Particles Containing a TLR9 Agonist Combined with Systemic αPD-1 Activate Tumor-Specific CD8+ T Cells
Source: Cancer Res Commun. 2026 May 1;6(5):1006–19. doi: 10.1158/2767-9764.CRC-26-0175 (PMC13133427; doi:10.1158/2767-9764.CRC-26-0175)
Supplement: Supplementary Figure S7 — Figure S7. The number of tumor-specific CD8+ T cells but not the number of total CD4+ T cells per gram of tumor increases with Vidu therapy. [file crc-26-0175_supplementary_figure_s7_suppsf7.pdf]

Supplemental Figure 7

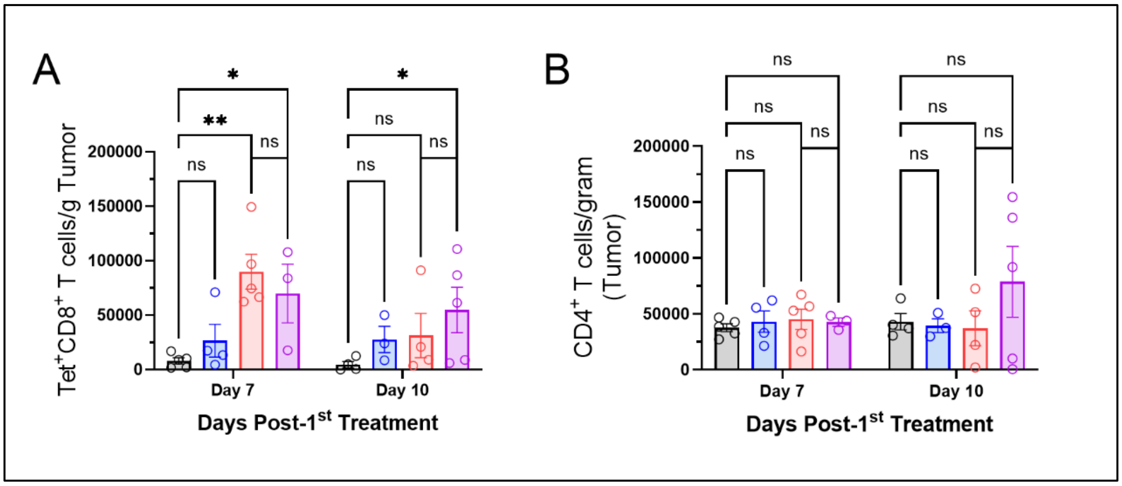

Supplemental Figure 7 The number of tumor-specific CD8$^+$ T cells but not the number of total CD4$^+$ T cells per gram of tumor increases with Vidu therapy. Wild Type C57BL/6 mice were primed with Vidu (day –31) followed by subcutaneous implantation with E.G7-OVA tumor cells (day –12) and adoptive transfer of tumor-specific CD8+ T cells via the retro-orbital sinus (day –1). Mice received IT injections of either saline or Vidu and IP injections of Isotype IgG or αPD-1 on days 0, 3, and 6 with an additional IP injection of Isotype IgG or αPD-1 on day 9. (A) Number of tumor-specific CD8$^+$ T cells per gram of tumor in Isotype/saline (black), αPD-1/saline (blue), Isotype/Vidu (red), and αPD-1/Vidu (purple) treated mice. (B) Number total CD4$^+$ T cells per gram of tumor in Isotype/saline (black), αPD-1/saline (blue), Isotype/Vidu (red), and αPD-1/Vidu (purple) treated mice. (n=3-5 mice/group)
